# Development and Characterization of Optimized Drug-Loaded Niosomes for Delivery of 5-FU and Irinotecan

**DOI:** 10.3390/pharmaceutics17070900

**Published:** 2025-07-11

**Authors:** Kafilat O. Agbaje, Simeon K. Adesina, Amusa S. Adebayo

**Affiliations:** Department of Pharmaceutical Sciences, College of Pharmacy, Howard University, 2300 4th Street NW, Washington, DC 20059, USA; kafilat.agbaje@bison.howard.edu (K.O.A.);

**Keywords:** niosomes, 5-fluorouracil, Irinotecan, biorelevant dissolution

## Abstract

**Background/Objectives**: 5-Fluorouracil (5-FU) and Irinotecan (IRT) are two of the most used chemotherapeutic agents in CRC treatment. However, achieving treatment goals has been hampered by poor drug delivery to tumor sites and associated toxicity from off-target binding to healthy cells. Though the synergism of 5-FU-IRT has provided incremental improvements in clinical outcomes, the short elimination half-life and off-target binding to healthy cells remain significant challenges. We postulated that nanoencapsulation of a combination of 5-FU and IRT in niosomes would prolong the drugs’ half-lives, while over-encapsulation lyophilized powder in Targit^®^ oral capsules would passively the CRC microenvironment and avoid extensive systemic distribution. **Methods**: Ranges of formulation and process variables were input into design of experiment (DOE Fusion One) software, to generate screening experiments. Niosomes were prepared using the thin-film hydration method and characterized by size, the polydispersity index (PDI), morphology and intrastructure, and drug loading. Blank niosomes ranged in size from 215 nm to 257 nm. **Results:** After loading with the 5-FU-IRT combination, the niosomes averaged 251 ± 2.20 nm with a mean PDI of 0.293 ± 0.01. The surfactant-to-cholesterol ratio significantly influenced the niosome size and the PDI. The hydrophilic 5-FU exhibited superior loading compared to the lipophilic IRT molecules, which probably competed with other lipophilic niosome components in niosomes’ palisade layers. In vitro dissolution in biorelevant media showed delayed release until lower intestinal region (IRT) or colonic region (5-FU). **Conclusions:** Thus, co-nanoencapsulation of 5-FU/IRT in niosomes, lyophilization, and over-encapsulation of powder in colon-specific capsules could passively target the CRC cells in the colonic microenvironment.

## 1. Introduction

Niosomes are vesicular drug delivery systems that have been widely used to deliver various types of drugs to diseased sites in the body. [1,2]. They are typically made from non-ionic surfactants such as sorbitan esters (Spans) and polysorbates (Tweens). Cholesterol is usually added to impact membrane stability, while diacetyl phosphate is commonly added to impart a charge to the niosomes [3,4]. The surfactants in a niosome are arranged such that the hydrophilic head group comes together to form a hydrophilic core while the hydrophobic tail groups assemble to form a hydrophobic layer [5]. The presence of both hydrophilic and hydrophobic spaces in niosomes makes them a versatile delivery system for drugs with a wide range of hydrophilicity and hydrophobicity [6].

Niosomes are nanovesicular colloidal systems ranging in size from large multilamellar vesicles to large unilamellar (100 to 3000 nm) and small unilamellar vesicles (10 to 100 nm) [7]. The use of niosomes as a drug delivery system offers several advantages such as ease of preparation, biocompatibility, versatility (they can encapsulate both hydrophilic and hydrophobic drugs), and low immunogenicity. Compared to liposomes, niosomes have been shown to have a longer circulation half-life, enhanced stability at room temperature, and minimal susceptibility to light and oxidation [8,9,10]. Niosomes are prepared in various ways such as the thin-film hydration method (TFH), the ether ejection method, the hand-shaking method, and the reversed-phase evaporation technique [11,12].

Niosomes are characterized based on their particle size, polydispersity index, zeta potential, morphology and structure, stability, and in vitro drug release [13]. Formulation variables such as the concentrations of surfactants and cholesterol used have been shown to have impacts on the characteristics of niosomes [14,15]. The effectiveness of niosomes as promising drug delivery systems for the management of cancer has been demonstrated by several studies. Chemotherapeutic agents such as doxorubicin, paclitaxel, and methotrexate have been encapsulated into niosomes for the management of various forms of cancers such as breast cancer and colorectal cancer [16,17].

5-Fluorouracil (5FU) and Irinotecan (IRT) are chemotherapeutic agents used in the treatment of colorectal cancer (CRC). They are used as first-line treatments for the management of stage 4 metastatic CRC and are the two major drugs in the combination regimen FOLFIRI, which is widely used clinically for the management of CRC [18,19]. According to the biopharmaceutical classification system (BCS), 5FU belongs to class III (high water solubility), while IRT belongs to class II (low water solubility) [20]. 5-FU has a very short half-life (8 min), while IRT has a fairly long t_1/2_ (6–12 h) [21,22]. The short half-life of 5-FU necessitates constant infusion of the drug to obtain the required amount at the target site and match the cytokinetic (growth cycle) profile of cancer cells. However, constant infusion over-exposes healthy cells to the cytotoxic drug, and a very small amount gets to the tumor site before the drug is cleared from the system, leading to therapeutic failure. In addition, continuous infusion is inconvenient for both patients and healthcare providers, overutilizes healthcare resources, and accentuates dose-related adverse drug reactions [23,24].

A dual drug-loaded niosomal formulation that can be administered orally would be beneficial in terms of ease of administration for the patient, specifically targeting cancer cells, delivering the right amount of the needed chemotherapeutic agent, and limiting the exposure of healthy cells to cytotoxic agents.

This study, therefore, sought to formulate and optimize a dual drug-loaded niosome delivery system containing 5FU and IRT. We hypothesized that the hydrophilic 5FU would be loaded in the hydrophilic core of the niosome, while the more lipophilic IRT would be loaded in the hydrophobic palisade layer.

## 2. Materials and Methods

### 2.1. Materials

Sorbitan monostearate (Span 60) and dicetyl phosphate were obtained from Sigma Aldrich (Allentown, PA, USA). Cholesterol was supplied by VWR (Swedesboro, NJ, USA), and DSPE-PEG-2000 was purchased from Nanosoft Polymer (Winston-Salem, NC, USA). The organic solvents used (methanol and chloroform) were from Sigma Aldrich (St. Louis, MO, USA) and were of analytical grade. Other materials used included a dialysis bag (Spectra/Por), an Allegra 64R centrifuge (Beckman Coulter, Brea, CA, USA), and formvar/carbon-coated 200-mesh copper grids (Electron Microscopy Sciences, Hatfield, PA, USA).

### 2.2. Methodology

#### 2.2.1. Design of Experiment

The proportions of niosome-formulation ingredients that yield optimal blank niosomes were determined using the design of experiment (DoE) software Fusion One version 6.1.8. Using ranges of input and output variables reported in the literature [25,26], a 2^5^ full factorial experiment was designed. DoE analysis produced a total of 32 plus 5 central points (a total of 37) for the experimental runs. The input and process variables and the resulting recommended experiments are shown in Table 1 and Table 2, respectively.

#### 2.2.2. Preparation of Niosomes

**Figure 1:**
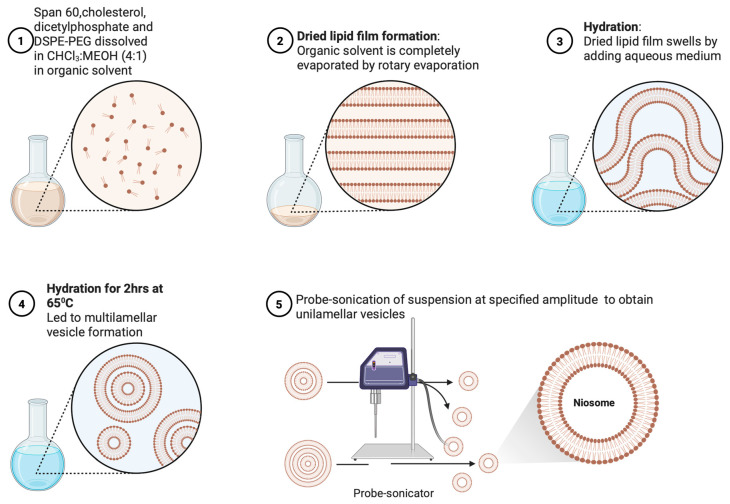
Illustrations of steps in preparation of niosomes. A surfactant (Span 60), cholesterol, dicetylphosphate, and DSPE-PEG were dissolved in a chloroform/methanol (4:1) mixture, and the organic solvent was removed by rotary evaporation to form a thin lipid film. Upon hydration with an aqueous medium at 60 °C for 2 h, multilamellar vesicles were formed. They were subsequently probe-sonicated to obtain unilamellar niosomes.

The niosomes were prepared using the thin-film hydration method (TFH). This preparation method is illustrated in Figure 1. Span 60, cholesterol, DSPE-PEG, and diacetyl phosphate were dissolved in chloroform/methanol (4:1). The solvent was removed using a rotary evaporator (Buchi Rotavapor R-100) at 100 rpm and 45 °C until a thin film was formed. 

The thin film was hydrated with phosphate-buffered saline (PBS) (pH 7.4) for 2 h on a rotary evaporator maintained at 65 °C and 125 rpm. The preparation was ultrasonicated using a probe sonicator (Sonics Vibracell, CT, USA) with a microtip with an amplitude of 20–35% for 5 min. The final product was centrifuged (Beckman Coulter Allegra 64R) at 120,000 rpm for 1 h. The supernatant was removed, and the resulting pellet was resuspended in PBS for further analysis. Illustrations of niosome formation and downstream processing are shown in Figure 1.

#### 2.2.3. Determination of Particle Size and Polydispersity Index

The particle sizes and size distributions of blank and drug-loaded niosomes were analyzed using a 90-plus particle size analyzer (Brookhaven Instruments Corp., New York, NY, USA). This analyzer used dynamic light scattering (DLS) to measure the hydrodynamic diameters of niosome suspensions [27]. Briefly, 200 uL of a sample was placed in a glass cuvette, which was filled to 2 mL with deionized water, and analyzed for particle size and the polydispersity index (PDI).

#### 2.2.4. Scanning Electron Microscopy (SEM)

Niosome morphology was examined by SEM analysis, as previously reported [25]. Briefly, to determine the size of the particles, all lyophilized compounds were mounted on aluminum stubs using carbon tape for SEM analysis. Imaging was performed using an in-lens FEI Teneo FEG SEM (ThermoFisher Scientific, Pittsburgh, PA, USA). High-resolution images were acquired under high-vacuum conditions with a voltage of 2 kV, a beam landing current of 25 pA, and a working distance of 3.9 mm. The horizontal field width was set to 11.8 µm, and the pixel size was 1.93 nm. A dwell time of 1 µs was used, along with a resolution of 6000 × 6000 pixels for each SEM image. No coating was applied during the SEM analysis.

#### 2.2.5. Transmission Electron Microscopy (TEM)

For 5 min, 5 mL of a nanoparticle suspension was applied to formvar/carbon-coated 200-mesh copper grids (Electron Microscopy Sciences) that were glow-discharged (20 mA for 60 s) right before use. EVs were negatively stained with 1% uranyl acetate (Electron Microscopy Sciences) for 2 min and air-dried before imaging. An FEI Talos F200X transmission electron microscope (Thermo Fisher Scientific, Waltham, MA, USA) operating at 200 kV was used. Images were acquired with a Thermo Scientific Ceta 16M CMOS camera.

#### 2.2.6. Statistical Analysis and Optimization of Blank Niosome

The particle size and PDI obtained from the screening experiment described above (Section 2.2.3) were input into the “Data Mining and Analysis” module of the DoE software, and the corresponding outputs (the particle size and PDI) were entered for the respective input variables (i.e., SP60, CHOL, DSPE-PEG, the hydration volume, and the sonicator amplitude). Various statistical parameters were examined, including “Non-linear Data”, “Experimental Error”, and “Regression Statistics”. The “Optimizer Wizard” was run, and the optimizer module was queried for the predicted optimized formulation for blank niosomes. The predicted optimized formulation (SP60, 75 mM; CHOL, 150 mM; DSPE-PEG, 10 mM; hydration volume, 15 mL; and sonicator amplitude, 30) was then prepared in triplicate, as described in Section 2.2.2 above. The niosomes’ particle size, PDI, and zeta potential were determined as previously described (Section 2.2.3) using a 90-plus particle size analyzer (Brookhaven Instruments Corp., NY, USA).

#### 2.2.7. Fourier Transform Infrared Spectroscopy (FT-IR) Analysis

FT-IR analysis was carried out to determine the interactions between the drug-loaded niosomes and the components of the niosome formulation. Samples of cholesterol, Span 60, 5FU, IRT, and drug-loaded niosomes were loaded into the sample chamber of a Spectrum 100 Fourier Transform Infrared (FTIR) spectrophotometer (Perkin Elmer, Shelton， CT, USA).

#### 2.2.8. Analytical Method Development

A reverse-phase HPLC method for quantitation of 5FU and IRT in niosomes was developed. The instrument comprised an Agilent series 1260 Infinity II HPLC system equipped with a Zorbax Eclipse plus C18 column, an Agilent autosampler (Series DE-23910399), a diode array detector, and Agilent OpenLab CDS analytical software Version 2.8 (Agilent Technologies, Santa Clara, CA, USA). The peak areas and retention times of both drugs were extracted from HPLC chromatograms. The mobile phases and analytical parameters were modified until the two samples were clearly and distinctly resolved, as indicated by their elution times and the quality of their chromatograms. The method that provided the best separation between 5FU and IRT was used for the rest of the analysis.

#### 2.2.9. Determination of Drug Loading Percentage

The optimized blank niosomes were prepared and loaded with 5FU and IRT. The thin-film rehydration (TFH) method, described above, was used with a slight modification. IRT was dissolved in the organic phase while a PBS hydration medium was replaced with a 5FU solution, and the thin film was hydrated for 2 h at 65 °C. The niosome dispersion was then sonicated. The resulting drug-loaded niosomes were centrifuged at 120,000 rpm for 30 min, the supernatant was removed, and the presence of unencapsulated drugs was assessed. The resulting pellet was washed thrice with PBS, dispersed in PBS, and freeze-dried to obtain a lyophilized niosome powder. The freeze-drying process was carried out by freezing the dispersion to about −20 °C, followed by drying under high vacuum at a temperature of −80 °C until a lyophilized powder was obtained.

A known amount of the drug-loaded niosomes was dissolved in acetonitrile, filtered into an HPLC vial using a 0.2 μm syringe filter, and analyzed for its drug content using the validated HPLC method described earlier (Section 2.2.8).

The percentages (%) of the drugs loaded into the niosomes were determined as follows:

(1) $$\%\;\mathrm{drug}\;\mathrm{loading}=\frac{Mass\;of\;drug\;loaded\;in\;niosome}{Mass\;of\;niosome}\;\times 100\;$$

#### 2.2.10. Determination of Encapsulation Efficiency

The loaded niosomes were centrifuged for 30 min at 120,000 rpm. The supernatant was removed, and the pellet was resuspended in PBS to repeat the centrifugation for another 30 min. The supernatant was combined, filtered through a 2 µm filter, and diluted with acetonitrile. The drug content was determined by a validated HPLC method, as described in Section 2.2.8 above.

The % of drug encapsulation (Encapsulation Efficiency, EE) was calculated using the following equation:

(2) $$EE=\frac{Amount\;of\;initial\;drug\;added\;to\;niosome-Amount\;of\;drug\;of\;drug\;in\;the\;supernatant}{Amount\;of\;initial\;drug\;added\;to\;niosome}\times 100$$

#### 2.2.11. Drug Release from Niosomes

The rate of drug dissolution from the niosomes was determined using the dialysis membrane (DM) method [28]. First, 10 mg of the drug-loaded niosomes were dispersed in PBS (pH 7.4) and placed in a dialysis bag. The dialysis bag was immersed in an Eppendorf tube containing 14 mL of PBS. The tube was clamped to a variable-angle tube rotator, which was rotated at 10 rpm and maintained at 37 °C in a constant-temperature oven. Aliquots of the release medium were taken at different time intervals and replaced with equal volumes of fresh PBS to maintain the sink conditions. The sample solution was diluted with acetonitrile, filtered through a 0.2 μm filter into an HPLC vial, and analyzed using the previously described (Section 2.2.8) validated HPLC method.

#### 2.2.12. Drug Release from Drug-Loaded Eudragit (Eudracap^®^) Niosome Capsules

Samples of drug-loaded niosomes were loaded into Eudracap^®^ colon capsules (test) and hard gelatin capsule shells (control). The drug release from the capsules was evaluated in a USP dissolution test apparatus (basket apparatus) rotating at 100 rpm. The bath temperature was maintained at 37 °C. Each capsule was placed in the basket and connected to the spindle of the rotating element. Simulated physiologic fluids were prepared as follows: Simulated gastric fluid was prepared by dissolving 3 g of NaCl in 1450 mL of deionized water, and the pH was adjusted to 1.2 with diluted HCl. Simulated intestinal fluid was prepared by dissolving 10.2 g of potassium phosphate monobasic and 3.75 g of sodium dodecyl sulfate in 1000 mL of deionized water, and the pH was adjusted to 6.8 with 1 N NaOH. At the start of each analysis, 900 mL of simulated gastric fluid was placed in a hemispherical vessel, and the sample-bearing basket was lowered into the medium. The apparatus was immediately operated at 100 rpm. At specified time intervals, samples were withdrawn from the bath, diluted with acetonitrile, filtered through a 0.2 μm filter into an HPLC vial, and analyzed using the validated HPLC method. Each sample withdrawn was replaced with an equal volume of medium to maintain the sink conditions. Dissolution was monitored for 2 h in simulated gastric fluid. At the end of 2 h, the simulated gastric fluid was siphoned out of the vessel and replaced with simulated intestinal fluid. Samples were taken and analyzed for an additional 2 h. The medium was then replaced with simulated colonic fluid, and dissolution was monitored for an additional 20 h to complete the 24 h test cycle. The % of drug release at each sample point was plotted against time to obtain the dissolution profiles.

## 3. Results

### 3.1. Design of Experiment and Experimental Runs

**Figure 2:**
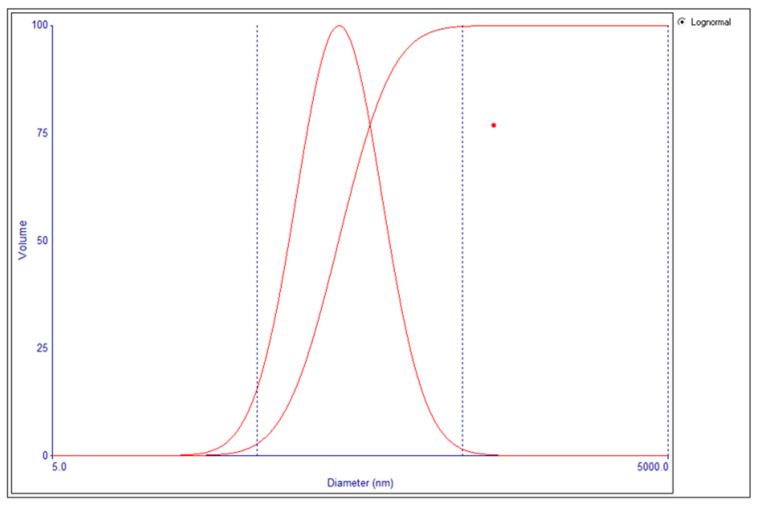
The particle size of a blank niosome. Effective diameter: 221.6 nm (±1.7 nm, SEM). Polydispersity index: 0.271 (±0.003, SEM). SEM = standard error of the mean. The particle size distribution of the blank niosomal formulation (normal = bell-shaped; logarithmic = labelled). The volume-based particle size distribution curve demonstrates a predominant population of particles with an effective diameter of 221.6 nm and a polydispersity index (PDI) of 0.275, indicating a relatively narrow size distribution. Measurements were conducted via dynamic light scattering at 25 °C, with an average count rate of 529.2 kcps. The inset table summarizes triplicate measurements, reporting consistent values across runs for the effective diameter, half-width, PDI, and baseline index. These results confirm the size uniformity of the niosomes.

The output variables accompanying each set of input variables in the experimental runs are presented in Table 3 below. A representative particle size distribution for the 37 formulations is shown in Figure 2. The mean particle size and PDI of the blank niosomes ranged from 215 to 257 nm and from 0.202 to 0.385, respectively. For the drug-loaded niosomes, the mean particle size and PDI were 251.5 nm ± 2.20, 0.293 ± 0.01, and −35.88 ± 2.96, respectively.

### 3.2. The Morphology of the Niosomes

**Figure 3:**
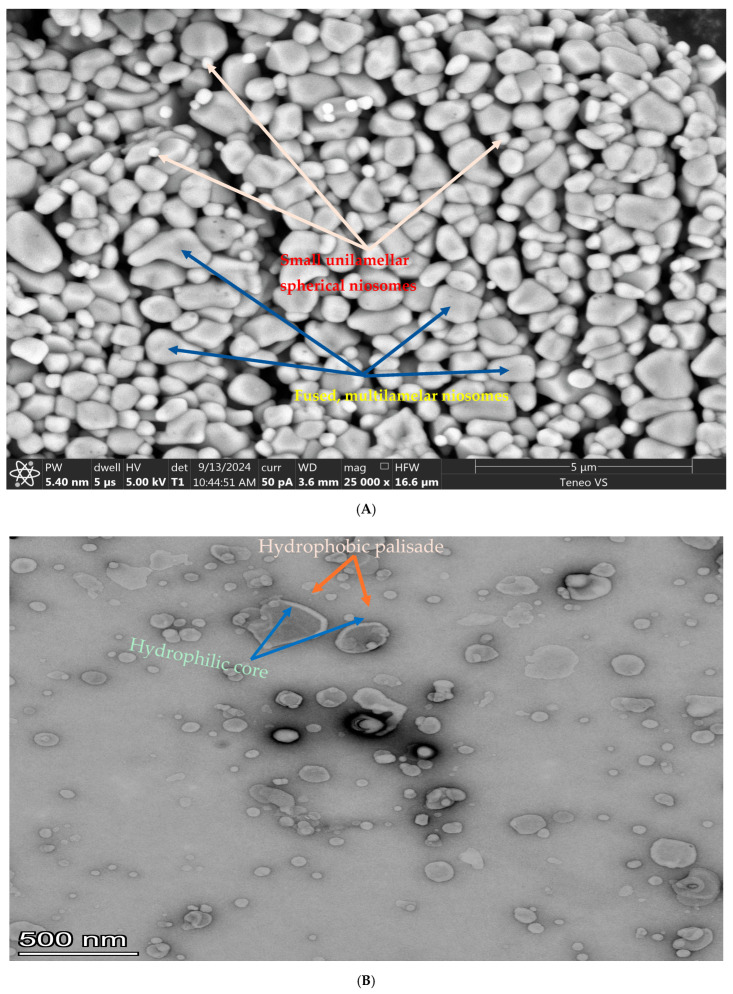
(**A**) A Scanning Electron Microscopy (SEM) image of lyophilized powder of the optimized 5FU-IRT-loaded niosome formulation. The niosome sphericity and uniformity characteristic of colloidal dispersion were modified by lyophilization. Small spherical unilamellar niosomes (red) and large fused multilamellar niosomes (yellow) are evident in the image. (**B**) A TEM image of a colloidal dispersion of the optimized 5FU-IRT-loaded niosome formulation. The TEM image highlights the internal structure of the niosomes, specifically highlighting the hydrophilic core and the hydrophobic bilayer of the vesicles.

An SEM image shows the spherical structure of the niosomes (Figure 3), while a TEM image provides information about the internal structure of the nanocarriers. The SEM image shows the morphology and gross surface structure of the niosomes, while the TEM image (with a much higher resolution) reveals the internal structure of the niosomes. The hydrophilic core and the hydrophobic layer of the niosomes are labeled in the TEM image below.

### 3.3. Statistical Analysis of Vesicle Size and PDI of Blank Niosomes

**Figure 4:**
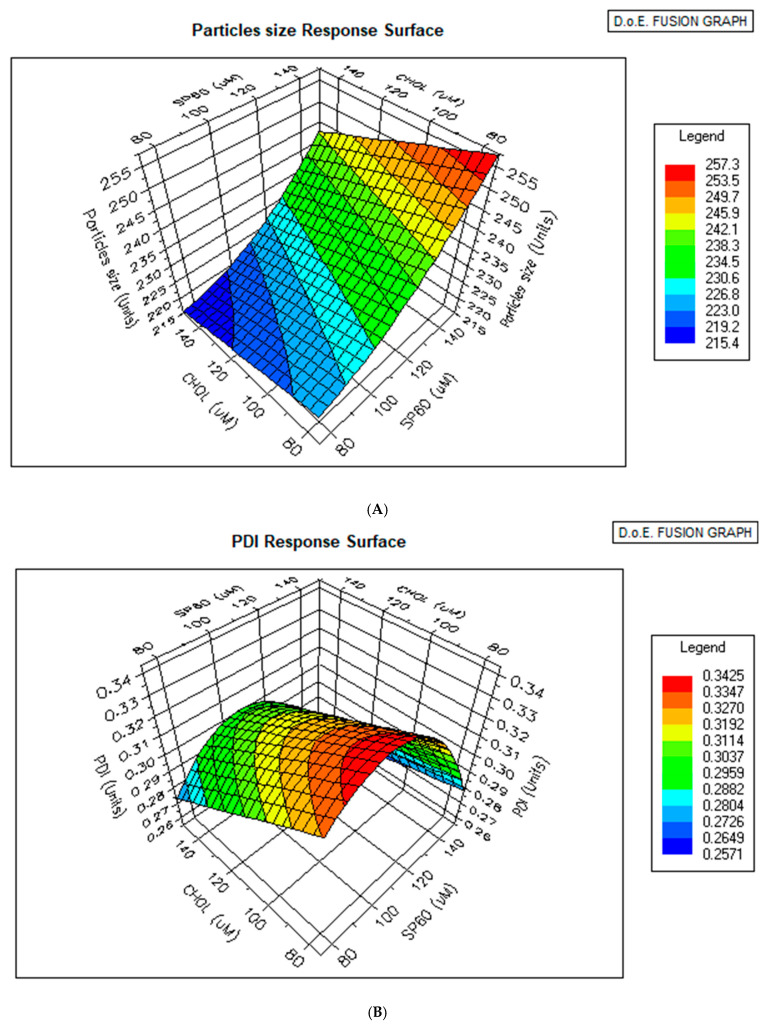
(**A**) A response surface plot showing the effects of the cholesterol (CHOL) and Span 60 concentrations on the particle size of the formulation. The 3D surface illustrates that particle size increased with increasing concentrations of both cholesterol and Span 60. At lower levels of CHOL and Span 60, smaller particle sizes (~215 nm) were observed, whereas higher concentrations of both excipients resulted in significantly larger particles (~257 nm). (**B**) A response surface plot illustrating the effects of the cholesterol (CHOL) and Span 60 concentrations on the polydispersity index (PDI) of the formulation. The 3D plot demonstrates a nonlinear relationship between the excipient concentrations and the PDI. At lower concentrations of both CHOL and Span 60, the PDI values were minimal (~0.26), indicating a more uniform particle size distribution. As the CHOL and Span 60 concentrations increased, the PDI initially rose, reaching a peak (~0.34), and then declined slightly at the highest levels of Span 60.

Coefficient tables for particle size and PDI are shown in Table 4 and Table 5. Each variable is ranked based on its contribution to the output. The variables that contributed most to particle size were Span 60 and cholesterol, while Span 60, cholesterol, and amplitude of sonication were significant determinants of PDI. *p* values < 0.005 represent statistical significance. Response surface graphics for particle size and PDI are shown in Figure 4, and they describe the relationship between the concentration of Span 60, the concentration of cholesterol, and particle size.

### 3.4. Optimizer Response

The algorithm-predicted formulation composition and process variable settings for the optimal vesicle size are shown in Table 6. The optimized niosomes were prepared in triplicate, and the particle size and PDI were determined. The particle size and PDI of the optimized formulation were 219 nm ± 3.64 and 0.244 ± 0.02, respectively, compared to the predicted values of 215.4 nm and 0.263.

### 3.5. Fourier Transform Infrared (FTIR) Analysis of Optimized Niosomes

**Figure 5:**
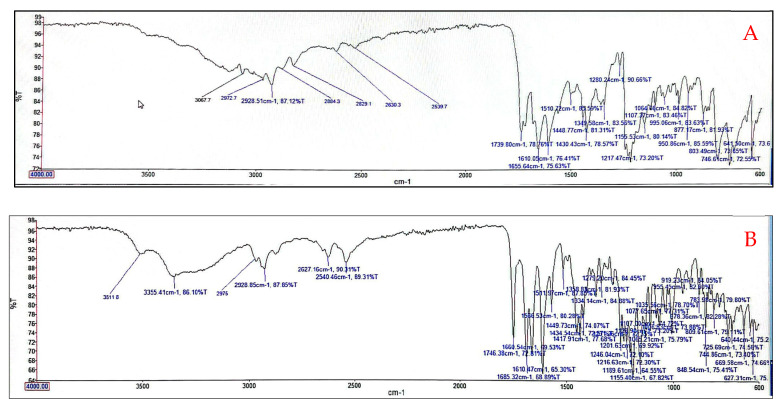

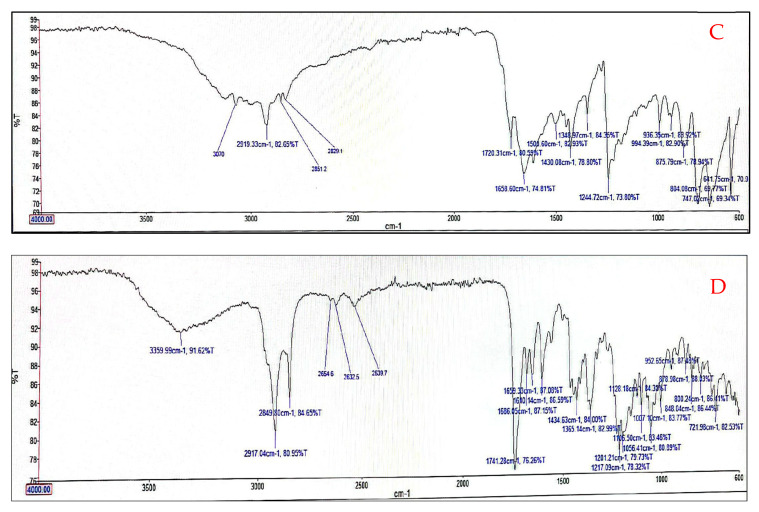
FTIR spectra of (**A**) 5-FU; (**B**) IRT; (**C**) SP60:CHOL (1:1); and (**D**) 5-FU-IRT-loaded niosomes. (**A**–**D**) show the FTIR spectra of 5-FU, IRT, SP60:CHOL (1:1) niosomes, and 5-FU-IRT-loaded niosomes, respectively. The spectrum of the blank niosomes (**C**) shows a broad -OH peak at 3359 cm^−1^, two prominent peaks at 2900–3000 cm^−1^ representing C-H stretching (aliphatic hydrocarbons), and a peak at 1700 cm^−1^ for C=O stretching. For the dual drug-loaded niosome (**D**), the features observed for the blank niosomes are also present, with additional low-intensity peaks at around 2500–2600 cm^−1^, representing C-H stretching, which is indicative of the presence of IRT. No peak relating to 5FU was observed because it was concentrated in the aqueous cores of the niosomes.

FTIR analysis of cholesterol, Span 60, blank niosomes, 5-FU, IRT, and drug-loaded niosomes was carried out, and spectra were obtained are shown in Figure 5A–D respectively. The spectra of the blank niosomes show a broad -OH peak at 3300 cm^−1^, two prominent peaks at 2900–3000 cm^−1^ representing C-H stretching (aliphatic hydrocarbons), and a peak at 1700 cm^−1^ for C=O stretching (Figure 5C). For the dual drug-loaded niosomes, the features observed in the blank niosomes (Figure 5C) are also present, with additional low-intensity peaks at around 2500–2600 cm^−1^, representing C-H stretching, which is indicative of the presence of IRT (Figure 5B). No peak relating to 5FU was observed in Figure 5D.

### 3.6. Analytical HPLC Method Development for Determination of Drug Loading and Encapsulation Efficiency

**Figure 6:**
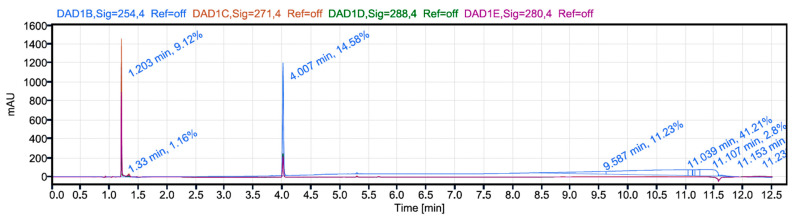
Sample chromatogram of 5FU and IRT from dual drug-loaded niosomes. 5-FU eluted at 1.2 min, while IRT eluted at 4.0 min. Best resolutions were obtained at 254 nm.

The analytical RP-HPLC method parameters that gave the optimal resolution for the 5FU and IRT chromatographic peaks are shown in Table 7. Figure 6 shows a typical chromatogram of 5FU and IRT from the dual drug-loaded niosomes. 5-FU eluted at 1.2 min, while IRT eluted at 4.0 min. The best resolutions were obtained at 254 nm. The validated HPLC method was used to assay analytical samples for the determination of the encapsulation efficiency (EE) and drug loading (DL).

The EE and DL percentages for 5FU and IRT were 72.3 ± 7.93 and 3.04 ± 0.08, and 68 ± 6.97 and 1.48 ± 0.06, respectively.

The HPLC chromatogram of the 5FU- and IRT-loaded niosomes (Figure 6) shows retention times of 1.2 min for 5FU and 4.0 min for IRT.

### 3.7. In Vitro Drug Release Studies

**Figure 7:**
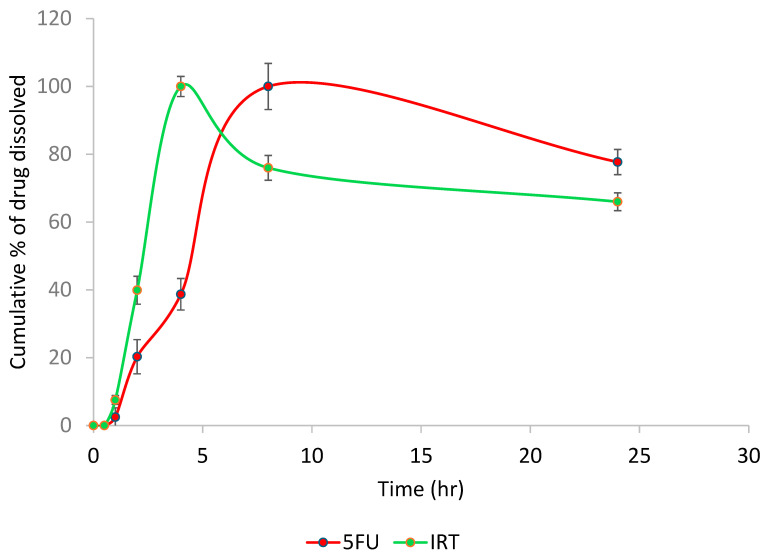
The in vitro drug release profiles of 5-fluorouracil (5FU) and Irinotecan (IRT) from the niosome formulation over 24 h. The graph presents the cumulative percentages of the drugs dissolved versus time for 5-FU (red line) and IRT (green line). Both drugs exhibited a biphasic release pattern. IRT showed a faster initial release, achieving over 90% release within 5 h, followed by a gradual decline. In contrast, 5FU displayed a more sustained release profile, with cumulative drug release increasing steadily and >90% release maintained until 24 h. The data are presented as means ± standard deviations, with the error bars representing the standard deviations.

The in vitro release of the drug-loaded niosomes was determined using the dialysis tube diffusion method. The dialysis membrane (DM) method is the most popular method to test the in vitro release of nanocarriers. The release medium used was phosphate-buffered saline (PBS). The cumulative release percentages of the two drugs are seen in Figure 7, which indicates no drug was released until after 1 h. In total, 100% of the 5-FU was released after 8 h, while 100% of the IRT was released after 4 h.

### 3.8. Drug Release Profiles of 5FU and IRT from Eudracap^®^ and Hard Gelatin Capsules

**Figure 8:**
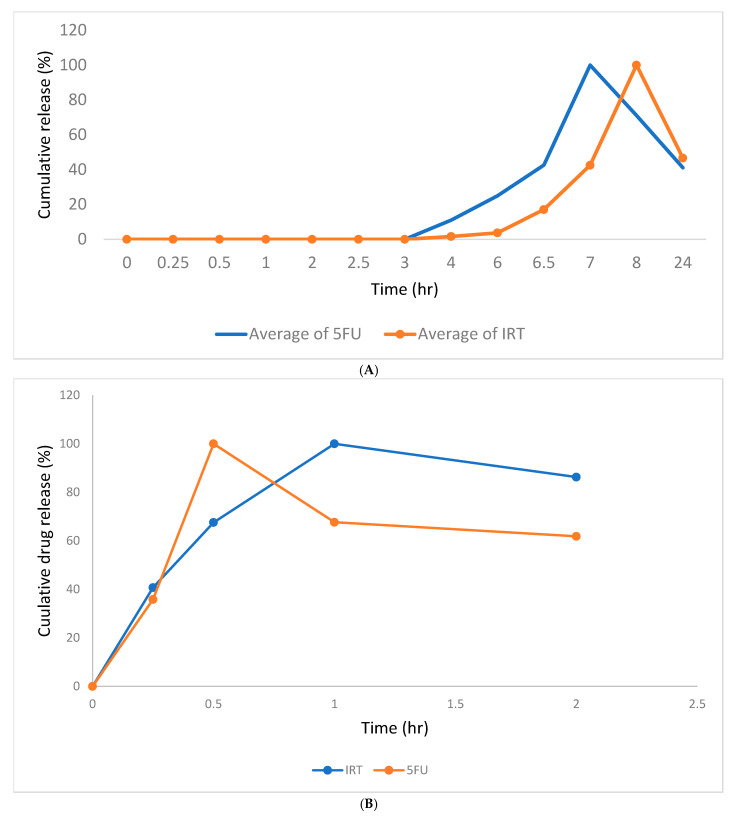
(**A**) The in vitro cumulative drug release profiles of 5-fluorouracil (5FU) and Irinotecan (IRT) from Eudragit^®^ capsules over 24 h in simulated gastrointestinal conditions. The dissolution study was conducted under sequential pH conditions representing different regions of the gastrointestinal tract: 0–2 h—simulated gastric region, 2–4 h—simulated intestinal region, and 6–24 h—simulated colonic region. IRT (blue curve) exhibited rapid drug release near the end of the intestinal region, followed by a gradual decline in the drug concentration in the colonic region. In contrast, 5FU (orange curve) showed slower release in the gastric and intestinal phases, with a sustained and controlled release profile observed predominantly in the colonic region, maintaining over 90% release until 24 h. The data are presented as means ± standard deviations, with the error bars indicating variability among replicates. (**B**) The in vitro cumulative drug release profiles of 5-fluorouracil (5FU) and Irinotecan (IRT) from conventional hard gelatin capsule formulations over 24 h in simulated gastrointestinal fluids. The dissolution study was carried out under sequential pH conditions simulating the gastrointestinal tract: 0–2 h—gastric, 2–4 h—intestinal, and 6–24 h—colonic. Both 5FU (orange curve) and IRT (blue curve) exhibited immediate and complete release within the first 2 h, corresponding to the gastric phase, with 5FU reaching peak release at 0.5 h and IRT reaching peak release at 1 h. This rapid release is attributed to the prompt disintegration of the gelatin capsules in the acidic environment, resulting in full release of both drugs before transitioning to the intestinal phase. No additional release was observed in the subsequent media. The data are expressed as means ± standard deviations, with the error bars representing intersample variability.

The drug release patterns of 5FU and IRT from a colon capsule, in comparison to the release from a hard gelatin capsule, are shown in Figure 8A,B below. Drug release from the Eudracap^®^ colon capsule started after about 4 h (indicating the terminal end of the small intestine), while 100% drug release was observed at 1 h for niosomes loaded into hard gelatin capsules.

## 4. Discussion

5-Fluorouracil (5-FU) and Irinotecan (IRT) are two of the most commonly used chemotherapeutic agents in colorectal cancer treatment. They are commonly employed as first-line treatments for various other cancers, including breast, head and neck, pancreatic, and stomach cancers. They are usually administered intravenously over a couple of days as part of a treatment regimen. Although 5-FU and IRT are effective chemotherapeutic agents for colorectal cancer (CRC), they have several limitations, such as rapid metabolism, a short half-life (especially for 5-FU), low bioavailability, high cellular toxicity, and poor selectivity for cancer cells. These factors reduce their overall effectiveness in cancer treatment. Rapid clearance of 5-FU from systemic circulation necessitates continuous intravenous infusion, which may overburden patients with high doses and inconveniences patients, healthcare providers, and other resources. To overcome these drawbacks, many researchers have focused on developing new delivery systems for 5-FU and IRT. These targeted drug delivery systems (DDSs) aim to minimize cytotoxic effects in healthy tissues while enhancing the impact on cancerous cells.

Among various carriers, niosomes have been widely used, owing to their ease of preparation, biocompatibility, physical stability, and cost-effectiveness. Niosomes are primarily made of non-ionic surfactants such as Span 60 and Tween 80. The non-ionic surfactants form an amphiphilic bilayer with the capacity to encapsulate hydrophilic drugs (in this case, 5FU) in their hydrophilic core and hydrophobic drugs (IRT) in their hydrophobic bilayer. The bilayers are usually stabilized with cholesterol, and charge-inducing agents are sometimes added. The proportions of non-ionic surfactants and cholesterol used in the formulation of niosomes have been shown to have significant impacts on properties such as the particle size, PDI, and encapsulation efficiency.

Achieving niosome formulations with small particle sizes requires evaluating various formulation and process variables to determine their effects on particle size. The traditional approach of adjusting one variable at a time is time-consuming and inefficient. Moreover, it fails to capture the interactions between different variables [29]. To address these challenges, researchers have adopted statistical experimental designs, such as the full factorial design. A full factorial design examines both the main effects and the interactions between independent and dependent variables by calculating the number of experimental runs needed to test all possible combinations of the independent variables at each level.

Particle size and the PDI are important parameters of niosomes because they determine both the physicochemical stability and biological fate of niosomes. Studies have shown that particles below the renal clearance threshold (about 10 nm) are rapidly filtered by the kidneys, while larger particles are prone to being easily recognized and cleared by the reticuloendothelial system (RES). It is therefore important to control the sizes of nanocarriers to prevent premature clearance and ensure therapeutic efficacy. Therefore, to maximize tumor accumulation and prevent renal clearance, the optimal nanoparticle size has been suggested to range between 10 and 300 nm.

In this study, the particle size indicates that the blank niosome formulation (215–257 nm) and the drug-loaded formulation (251 ± 2.20 nm) fall within the acceptable range to enable tumor accumulation and evade renal clearance. The polydispersity index (PDI) of nanocarriers is the degree of dispersity of particles in a sample and is a crucial parameter for nanoparticles [30]. It measures the distribution of the particle sizes within a niosome sample. PDI values range from 0 to 1, with lower values indicating a more uniform or monodispersed particle size distribution [31]. The low PDI of the formulated niosomes (0.293 ± 0.01) indicates that the particles are uniformly dispersed with great potential for product stability.

The zeta potential of nanocarriers determines the surface charge, which determines how nanocarriers interact with the biological environment and their electrostatic interactions with bioactive compounds. High zeta potential values (either positive or negative) ensure stability and prevent aggregation of particles via electrostatic repulsion [32]. The zeta potential of the drug-loaded niosomes in this study is negative, suggesting that this formulation is stable.

The response graphics obtained during this study show that the particle size decreases as the concentration of cholesterol increases. This explains why the optimizer response predicted that minimization of particle size would be achieved using the highest concentration of cholesterol. This result is in accordance with data in the literature. In 2015, Mavaddati et al. [33]. stated that the vesicle size of niosomes decreases as the concentration of cholesterol increases from 25 to 50 mol%. The effect of cholesterol on the particle size of niosomes has been shown to be dependent on the type of surfactant used. For Span 60 niosomes with low HLB values, increasing the amount of cholesterol causes a significant decrease in the average particle size [34]. This is because cholesterol can align parallel to the hydrocarbon chains of amphiphilic surfactants. The hydroxyl group of the sterol moiety of cholesterol forms a hydrogen bond with the ester group in non-ionic surfactants. Thus, cholesterol can enhance the bilayer hydrophobicity, leading to a decrease in the surface free energy and therefore a particle size reduction [35].

The FTIR spectra of the blank niosomes and drug-loaded niosomes reveal characteristic signals associated with Span 60 and cholesterol in the range of 3500–3000 cm^−1^ (Figure 5). These signals appear broader due to the intermolecular interaction generated by a hydrogen bond between SP60 and cholesterol. A characteristic signal of stretching and flexion of the hydroxyl group is located at 3359 cm^−1^, and there is another signal at 2935 cm^−1^, corresponding to the C-H stretching of the methyl (CH3) cholesterol groups. A characteristic C-O signal is present in the range of 1695–1741 cm^−1^, corresponding to the ester bond. The signal at 1434 cm^−1^ corresponds to the symmetrical stretching of the carbonyl group of SP60. The absence of any strong new signals between the niosome components and the encapsulated drugs indicates that the niosomes maintain their integrity as nanocarriers, as well as compatibility between the drugs and the niosome components.

The drug loading of 5FU was 50% higher than that of IRT. The disparity in the loading of the two drugs was due to the presence of other competing factors in the bilayer. In the hydrophobic bilayer, cholesterol, IRT, and diacetylphosphate all compete for the available space, which reduces the drug loading of IRT, unlike 5FU, which loads in the hydrophilic core, where it has no competition.

The analytical HPLC employed a reverse-phase C-18 column (5 µm, 4.6 × 150 mm). The column temperature was kept at 37 °C, and the mobile phase was acetonitrile and water acidified with 1% TFA. The analysis was performed via gradient elution, and there was proper separation of the two drugs, as 5FU had an average retention time of 1.25 (±0.54) min and IRT had an average retention time of 3.85 (±0.62) min. As expected, based on its hydrophilicity, 5FU eluted from the column first, followed about 3 min later by the hydrophobic IRT (Figure 6).

The drug release profile is a critical parameter in drug delivery systems. It constitutes the rate-limiting step in the systemic or site-specific appearance of a nanoencapsulated drug. In vitro studies were conducted in simulated or biorelevant physiological conditions to have an idea of how the proposed system would behave in in vivo conditions [36,37]. The in vitro drug release profile of the 5FU- and IRT-loaded niosomes showed that IRT was released faster than 5FU. This was expected because the IRT, being in the hydrophobic bilayer, was closer to the release medium than the 5FU, which was in the hydrophilic core. The release of IRT at 4 h and 5FU at 8 h is an indication that the two drugs will get to the site of action before release occurs, thereby overcoming the problem of premature release peculiar to some drug delivery systems.

Lyophilized powder of the drug-loaded niosomes was loaded into a Eudracap^®^ colon-targeted capsule (test) and a conventional hard gelatin capsule (control). The dissolution media simulated differing pH conditions throughout the gastrointestinal tract. For the Eudracap^®^ colon capsule, no drug release was observed during the first 2 h, when the capsules were in a pH range reflective of the gastric region. In the case of the hard gelatin capsule, however, drug release started after approximately 30 min in the gastric region and peaked at 1 h. The absence of drug release from the Eudracap^®^ capsule in the early hours demonstrates the reliable acid-resistant nature of the capsule. As the conditions changed for the simulated intestinal environment, drug release was observed in the terminal section of the intestinal region, increasing gradually as the conditions changed for the colonic region. In total, 100% drug release was observed in the third hour in the colonic region. These observations indicate that the hard gelatin capsule offers little to no protection for formulations and can lead to premature release of the cytotoxic agent. The Eudracap colon-targeted capsules, on the other hand, effectively protected the niosome formulation until it reached the colonic region, reiterating its usefulness in colon-specific drug delivery.

## 5. Conclusions

In this study, dual-drug niosomal formulations of 5FU and IRT were developed and optimized. The optimized drug-loaded niosomes had an average size of 251.5 ± 2.20 nm and a mean polydispersity index (PDI) of 0.293 ± 0.01. The hydrophilic BCS class III compound (5FU) exhibited a superior (about double) loading capacity compared to the lipophilic BCS class II compound (IRT). It appears that lipophilic drug molecules compete with other lipophilic components of niosomes, which limits their residence in the palisade layers of niosomes, thereby limiting their loading capacity.

The analytical HPLC method was sensitive and specific, and it was able to resolve 5FU and IRT in a single, multi-channel reverse-phase HPLC run. Although the literature reported detection of IRT at 280 nm and detection of 5FU at 254 nm, the two drugs were more quantitatively resolved at 254 nm. The mean retention times were 1.25 ± 0.54 min for 5FU and 3.85 ± 0.62 min for IRT.

FTIR analysis indicated neither physical–chemical interactions between the formulation ingredients and the drugs nor process-induced alterations to the molecular nature of the formulation components. Also, the Eudracap^®^ colon-specific capsule effectively protected the formulation, with no drug release during the first 2 h, when the capsules were in a pH range reflective of the gastric region. Drug release from the Eudracap^®^ capsules started at about 4 h (reflective of the pH and the transit time to the proximal end of the small intestine), while 100% drug release from niosomes loaded into hard gelatin capsules occurred at about 1 h. Thus, the Eudracap^®^ capsules effectively protected the loaded niosomes from being released in the upper GIT. Release started after 2 h in intestinal fluid, indicating potential for loaded drugs to begin release in a region of the GIT proximate to the colonic region. On the other hand, the dissolution from the hard gelatin capsules was consistent with that of conventional capsules, showing 100% drug release in 1 h. There is, therefore, potential for Eudracap^®^ capsules to passively direct niosome-loaded drugs to the colonic cellular microenvironment of CRC.

## Figures and Tables

**Table 1:** The variable levels input into the DoE platform.

Independent Variables	Levels	Dependent Variables
Span 60 concentration	75–150 µM	Particle size Polydispersity index
Cholesterol concentration	75–150 µM	
DSPE-PEG concentration	2–10 µM	
Hydration volume	10–15 mL	
Amplitude of sonication	20–30%	

**Table 2:** Layout of experiment and output variables.

	CHOL	Sp60	PEG-2000	Hydration Volume	Amplitude
Run No.	(µM)	(µM)	(µM)	(mL)	(%)
1	113	113	6	12.5	25
2	113	113	6	12.5	25
3	150	75	10	10	20
4	75	150	2	10	30
5	150	150	10	15	20
6	75	75	10	15	30
7	113	113	6	12.5	25
8	75	150	10	10	30
9	113	113	6	12.5	25
10	150	150	2	10	20
11	75	150	10	15	20
12	150	75	2	10	30
13	150	75	2	15	20
14	150	75	10	10	30
15	75	150	2	15	30
16	75	150	2	10	20
17	75	75	10	10	30
18	75	150	10	10	20
19	75	75	10	10	20
20	150	150	2	15	30
21	150	75	2	15	30
22	150	150	10	10	30
23	75	75	2	15	20
24	113	113	6	12.5	25
25	75	75	2	10	20
26	75	150	2	15	20
27	75	75	2	10	30
28	150	150	10	15	30
29	150	150	10	10	20
30	150	75	2	10	20
31	150	75	10	15	20
32	150	75	10	15	30
33	75	75	2	15	30
34	150	150	2	10	30
35	75	75	10	15	20
36	150	150	2	15	20
37	75	150	10	15	30

**Table 3:** Experimental runs and corresponding output variables.

	Formulation (Input) Variables			Processing Variables		Output Variables	
	CHOL	Sp60	PEG-2000	Hydration Volume	Amplitude	Vesicle Size	Polydispersity Index
Run No.	(μM)	(μM)	(μM)	(mL)	(%)	(nm)	
1	113	113	6	12.5	25	224.1	0.309
2	113	113	6	12.5	25	230.3	0.301
3	150	75	10	10	20	217.1	0.385
4	75	150	2	10	30	246.1	0.294
5	150	150	10	15	20	225.0	0.34
6	75	75	10	15	30	225.6	0.29
7	113	113	6	12.5	25	233.1	0.306
8	75	150	10	10	30	252.0	0.299
9	113	113	6	12.5	25	229.2	0.2315
10	150	150	2	10	20	243.3	0.333
11	75	150	10	15	20	257.4	0.279
12	150	75	2	10	30	218.2	0.341
13	150	75	2	15	20	220.7	0.333
14	150	75	10	10	30	216.4	0.311
15	75	150	2	15	30	254.6	0.254
16	75	150	2	10	20	230.1	0.254
17	75	75	10	10	30	221.8	0.202
18	75	150	10	10	20	238.6	0.254
19	75	75	10	10	20	228.6	0.264
20	150	150	2	15	30	242.4	0.358
21	150	75	2	15	30	227.9	0.324
22	150	150	10	10	30	243.5	0.206
23	75	75	2	15	20	222.9	0.278
24	113	113	6	12.5	25	227.1	0.313
25	75	75	2	10	20	221.6	0.275
26	75	150	2	15	20	232.3	0.274
27	75	75	2	10	30	224.7	0.268
28	150	150	10	15	30	221.5	0.339
29	150	150	10	10	20	239.2	0.35
30	150	75	2	10	20	242.7	0.32
31	150	75	10	15	20	215.7	0.358
32	150	75	10	15	30	218.5	0.364
33	75	75	2	15	30	220.6	0.214
34	150	150	2	10	30	242.6	0.362
35	75	75	10	15	20	221.2	0.289
36	150	150	2	15	20	239.9	0.356
37	75	150	10	15	30	252.9	0.294

**Table 4:** Coefficient table and model for particle size.

Variable Name	Coefficient Value	Standard Error	t-Statistic	*p*-Value
Constant	229.719	1.370		
X1	14.984	0.541	27.678	<0.0001
X2	−5.972	0.541	−11.031	<0.0001
(X1)2	5.159	1.473	3.502	0.0014
X1∗X2	−1.478	0.541	−2.730	0.0102

Key: X1 = SP60, X2 = CHOL.

**Table 5:** Coefficient table and model for polydispersity index.

Variable Name	Coefficient Value	Standard Error	t-Statistic	*p*-Value
Constant	0.31908	0.00499		
X1	−0.01626	0.00201	−8.099	<0.0001
X2	−0.09105	0.00201	−9.485	<0.0001
X5	−0.00439	0.00201	−2.186	0.0371
(X1)2	−0.03512	0.00538	−6.534	<0.0001
X1∗X2	0.00849	0.00201	4.226	0.0002
XI∗X4	0.0439	0.00201	2.186	0.0371

Key: X1 = SP60, X2 = CHOL, X4 = hydration volume, X5 = amplitude of sonication.

**Table 6:** Algorithm-predicted formulation and process variable settings for optimal vesicle size.

Variable Name	Optimizer Specification for Variable Setting
SP60	75 µM
CHOL	150 µM
DSPE-PEG	10 µM
HYDVOL	15 mL
AMPL	30%

**Table 7:** The HPLC parameters for the resolution of 5FU and IRT.

Parameters	Value
Column type and dimension	C18 column (4.6 × 150 mm)
Column temperature	37 °C
Flow rate	1 mL/min
Injection volume	10 μL
Run time	16 min
Mobile phase	Water and acetonitrile (gradient elution)
Detection wavelength	254 nm

## Data Availability

The data presented in this study are available on request from the corresponding author due to related studies that are ongoing and pending patent applications.

## Abbreviations

The following abbreviations are used in this manuscript:

HPLC	High-Pressure Liquid Chromatography
EE	Encapsulation Efficiency
BCS	Biopharmaceutics Classification System
CHOL	Cholesterol
DCP	Dicetylphosphate
SAA	Surface-Active Agent
SEM	Scanning Electron Microscope
TEM	Transmission Electron Microscope
PDI	Polydispersity Index
